# Emotional Labour and Wellbeing: What Protects Nurses?

**DOI:** 10.3390/healthcare4040089

**Published:** 2016-11-30

**Authors:** Gail Kinman, Sandra Leggetter

**Affiliations:** 1 School of Psychology, University of Bedfordshire, Luton, Bedfordshire, LU1 3JU, UK; 2 School of Nursing, University of Bedfordshire, Luton, Bedfordshire, LU1 3JU, UK; sandra.leggetter@beds.ac.uk

**Keywords:** emotional labour, compassion, burnout, social support, coping

## Abstract

Although compassionate care has wide-ranging benefits for patients, it can be emotionally demanding for healthcare staff. This may be a particular problem for those with little experience in a caring role. This study utilises the job demands-resources model to examine links between “emotional labour” and emotional exhaustion in student nurses. In line with the triple-match principle—whereby interactive effects are more likely when job demands, resources, and outcomes are within the same qualitative domain—the protective role of emotional support and emotion-focused coping (i.e., emotional venting) in the relationship between emotional labour and exhaustion is also explored. An online questionnaire was completed by 351 student nurses with experience working in healthcare settings. A strong positive relationship was found between emotional labour and emotional exhaustion, and some support was found for the moderating effects of emotional support and emotion-focused coping. Ways to help student and qualified nurses develop the emotional resilience required to protect their wellbeing, while providing high-quality compassionate care to patients are considered.

## 1. Introduction

Compassion has been defined as a deep awareness of the suffering of another person, together with the desire to relieve it [1]. The provision of compassionate care is fundamental to the work of healthcare professionals, and has been found to be genuinely therapeutic for patients [2,3]. Although ratings of clinical competence by patients are typically based on a combination of experiential knowledge and technical skills [4], the quality of interpersonal relationships makes a strong contribution to judgements of overall satisfaction with healthcare experiences [5,6]. Indeed, an “uncaring” attitude from staff is one of the most frequent causes for complaints from patients and their families about the quality of service received [7,8]. A lack of care and compassion in healthcare settings can have grave consequences. Recent investigations into major failures of hospital care in the UK concluded that a lack of compassion and respect from staff made a strong contribution to patient neglect and suffering [9]. The key role played by nurses, in particular, in building a more compassionate, patient-centred culture has been emphasised [10].

### 1.1. Benefits and Drawbacks of Delivering Compassionate Care

A body of research has highlighted the wide-ranging benefits of compassionate care for service users. Healthcare practitioners who are considered to be more compassionate and empathic gain more trust from their patients who, in turn, tend to be more compliant, experience less pain and anxiety, and have a more favourable prognosis [11]. Building caring and compassionate relationships with patients can not only improve patient outcomes, but also enhance the wellbeing of practitioners themselves. There is evidence that compassion satisfaction gained from the helping role can protect them from work-related stress and burnout [12]. Moreover, empathic interactions with service users can engender feelings of personal accomplishment, replenish motivation, commitment, and energy, and build emotional resilience [11,13].

Although working in healthcare can be rewarding and satisfying, it can be emotionally demanding and stressful. UK health and safety statistics indicate that nurses are at greater risk of work-related stress, anxiety, and depression than other occupational groups [14]. Moreover, an international study [15] reported that 42 percent of a large sample of nurses from the UK described themselves as “burned out”. The emotional demands of healthcare work make strong contributions to the high levels of stress observed in the profession, as well as to compassion fatigue and burnout [11,16,17]. Attempts to distance oneself emotionally as a form of self-protection against feelings of emotional depletion is a key feature of burnout [18], but this can have negative implications for the wellbeing of practitioners and the quality of care [19].

The concept of emotional labour has been used to gain insight into the nature and impact of delivering compassionate care in healthcare contexts. This is discussed further in the next section.

### 1.2. Emotional Labour

“Emotional labour” refers to the effort involved in managing feelings when the work role specifies that particular emotions should be displayed and others should be hidden [20]. Jobs requiring emotional labour have three elements: (a) intensive contact with the public; (b) the need to produce an emotional state in other people; (c) a set of explicit or implicit rules regarding the type of emotional display that is appropriate and inappropriate [21]. As the “appropriate” emotional response will not always arise spontaneously, employees are required to suppress emotional reactions that are unsuitable for the job role (such as frustration and disgust) and display those (such as patience and empathy) that are more congruent [22]. Emotional dissonance—or the conflict between emotions that are experienced and those that are required to conform to display rules—is a source of strain that can threaten the wellbeing of employees and lead to burnout via the depletion of emotional resources [22,23].

Most public-facing jobs require some degree of emotional labour, but it is thought to be fundamental to nursing [24,25,26]. Nurses have sustained and intensive contact with patients and—as discussed previously—are required to deliver compassionate care often under challenging interpersonal conditions [27,28]. They are not only expected to regulate their own emotional reactions to practice, but also to assuage the fear and distress of their patients [29]. Moreover, nursing has a set of shared “display rules” concerning the emotions that should be expressed and those that should remain hidden [30]. Although performing emotional labour can be a source of satisfaction when performed for philanthropic reasons [31], it can threaten the wellbeing of healthcare workers [25]. For example, daily diary research suggests that nurses who have difficulty managing the emotional demands of the job tend to report elevated levels of negative affect, emotional exhaustion, and general fatigue [32].

There is evidence that early career nurses are likely to react more negatively to the emotional demands of practice and are at high risk of stress and burnout [33,34]. The need for enhanced training and support to help them manage the emotional demands of the job role has been recognised. To inform such interventions, insight into the implications of emotional labour and the factors that might protect inexperienced nurses from the negative effects of the emotional demands they encounter is required [35]. This study utilises the job demand–resources model (JD-R: [36]) to examine relationships between emotional labour and emotional exhaustion and the contextual and individual characteristics that might help them manage emotional demands more effectively.

### 1.3. The Job Demands-Resources Model

The JD-R model postulates that, while job demands can impair the wellbeing of employees, job resources have the potential to offset their negative impact [36]. Demands are the features of jobs that require sustained physical and/or psychological effort and, as such, can deplete energy levels and threaten wellbeing. Conversely, job resources are factors intrinsic to the individual or their environment that can enable them to fulfil the demands of their work and, accordingly, enhance their wellbeing. This model has been supported in studies within healthcare environments, whereby staff with fewer resources (such as autonomy and opportunities for feedback) were at greater risk of burnout derived from workload and patient demands [37].

### 1.4. The Matching Hypothesis

The present study conceptualises emotional labour as a job demand (i.e., the experience of emotional dissonance) that has the potential to lead to emotional exhaustion (i.e., feelings of being emotionally overextended and depleted). The role played by two potential resources—emotional support and emotion-focused coping—in this relationship are explored. These variables were selected on the basis of the matching principle that was first proposed by Cohen and Wills [38], whereby resources are considered more likely to protect wellbeing if they are from the same qualitative dimension as demands. Domains can be physical, cognitive, or emotional, and the greater the congruence between the demands and the potential resource, the stronger the protective effects that will be observed. The triple match principle [39] extends this hypothesis by maintaining that the probability of a moderating effect is further enhanced when demands, outcomes, and resources are within the same domain. Some evidence to support this principle has been found in studies of several occupational groups (including healthcare workers), whereby the likelihood of buffering effects was directly related to the degree of fit between demands, resources, and strain [40,41,42,43]. In a study of Canadian nurses [44], evidence emerged to support a triple-match; when emotional demands were high, nurses with fewer emotional resources tended to report more anxiety and depression. The present study extends previous research by testing the triple-match principle within the emotional domain in a sample of student nurses: more specifically, it is proposed that emotional resources (i.e., emotional support and emotion-focused coping) can mitigate the negative impact of emotional labour on emotional exhaustion.

### 1.5. Emotional Support and Emotion-Focused Coping

The key role played by social support in building emotional capacity and mitigating the effects of stressors on physical and mental health has been highlighted [45,46]. Of particular relevance to the present study is evidence that support from managers and colleagues can protect healthcare workers from the negative impact of emotional demands [7,27]. For example, a two-phase study of Australian nurses found that their capacity to cope with stress and compassion fatigue was enhanced by support gained from strong social networks [47]. Several types of social support have been documented that have been found to benefit the wellbeing of employees via multiple pathways; these include informational, instrumental, and professional support [48].

It has been argued that emotional support—characterised by the availability of close and confiding relationships within the workplace—may be particularly advantageous in jobs that are emotionally challenging [42]. Several explanations for such effects within healthcare settings could be proposed. The ability to disclose personal concerns and difficulties to others without fear of judgement may directly reduce the risk of emotional exhaustion and/or act as a resource that helps nurses manage the emotional demands of delivering compassionate care. Emotional support from more experienced managers and co-workers may also help student nurses invoke the appropriate emotional response required during interactions with patients, thus reducing experiences of emotional dissonance.

Coping can be defined as “constantly changing cognitive and behavioural efforts to manage specific external and/or internal demands that are appraised as taxing or exceeding the resources of the person” ([49]; p. 19). Coping strategies are broadly considered to be either emotion-focused or problem-focused. Emotion-focused coping aims to reduce the intensity of emotions caused by a stressful situation, whereby problem-focused coping attempts to change the problematic situation itself. Although there is evidence that nurses frequently utilise emotion-focused coping strategies to manage work-related stress [50], the implications for wellbeing are mixed and negative, and positive effects on mental health have been reported [51,52,53,54].

Various types of emotion-focused coping strategies have been documented, such as avoidance, minimisation, rumination, distancing, and gaining positive value from negative events [55]. The current study examines the role played by venting, or emotional expression. Emotional catharsis has long been considered beneficial for health, whereas the repression or inhibition of emotions has been linked with a range of negative outcomes [56]. There is evidence that emotional expression can help people process their emotional reactions, facilitate emotional adjustment, and enhance mental health and life satisfaction [57,58]. There is also evidence that nurses who use emotional expressive coping styles are at lower risk of emotional exhaustion [54]. Emotional venting may help nurses manage the emotional demands of their work for several reasons. It may help employees fulfil the emotional display requirements of their job role [59]. Emotionally expressive coping may be particularly beneficial in work requiring a high level of emotional labour, as opportunities to express genuine emotions—rather than merely comply with display rules—may protect wellbeing.

### 1.6. Aims of the Study

This study utilises the JD-R model to examine associations between emotional labour and emotional exhaustion in nurses. In line with the triple-match principle, the protective role of emotional support and emotion-focused coping in this relationship is also explored. Focus is placed on the experiences of student nurses. There is evidence that they find their clinical placements emotionally challenging and—as discussed previously—a lack of experience in the nursing role can exacerbate the negative impact of emotional labour on strain. It is therefore envisaged that the findings of this study will have the potential to inform interventions to help student nurses provide compassionate care without compromising their wellbeing.

## 2. Methods

### 2.1. Participants

An online questionnaire was completed by 351 student nurses (92% female) who were registered across three campuses of a university in the south-east of England. Participation was invited by email, and there was a 48 percent response rate. Forty-eight percent of participants were in their first year of study, 25 percent in their second year, and 27 percent in their third and final year. All participants had experience working in clinical areas. Ages ranged from 18 to 55, with a mean of 30.50 (*SD* = 9.36). Eighty-eight percent of the sample were UK nationals and the majority (74%) identified themselves as White British (74%). Participants provided informed consent.

### 2.2. Measures

A series of scales was used to obtain data. Higher scores on each of the measures represented higher levels of the construct being measured.

#### 2.2.1. Emotional Labour

Emotional labour was assessed by an adapted version of a five-item scale developed by Zapf et al. [60]. This assesses the extent to which respondents experience dissonance between emotions that are felt and those that they feel are required as part of the job role. Responses are obtained on a five-point scale ranging from “very rarely/never” to “always”. An example item is “When dealing with patients, I have to display emotions that do not agree with my true feelings” (Cronbach’s alpha = 0.92).

#### 2.2.2. Emotional Exhaustion

Emotional exhaustion was measured by the seven-item scale from the Maslach Burnout Inventory [18]. Items are rated on a seven-point scale ranging from 0 = “never” to 6 = “always”. An example item is “I feel emotionally drained from my work” (Cronbach’s alpha = 0.84).

#### 2.2.3. Emotional Support

Emotional support was assessed by a scale developed for the current study. This measured the extent of satisfaction with emotional support provided by several work sources, such as fellow students, work colleagues, and tutors. Responses were obtained on a five-point scale where 1 = “very dissatisfied” and 5 = “very satisfied” (Cronbach’s alpha = 0.89).

#### 2.2.4. Emotion-Focused Coping

Emotion-focused coping was assessed by the four-item venting/expressing emotion subscale from Carver et al. [61]. Respondents were asked to rate the extent to which they use each of the strategies on a four-point scale ranging from 1 = “I usually don’t do this at all” to 4 = “I usually do this a lot”. An example of an item is “I feel emotional distress and express these feelings” (Cronbach’s alpha = 0.86).

### 2.3. Procedure

A link to an online survey was sent by email to student nurses studying in three universities in the south-east of England. The study complied with the ethical requirements of the British Psychological Society, and was approved by the institution’s ethics committee.

## 3. Results

Correlations between study variables are provided in Table 1, together with means and standard deviations. The practical significance of the findings can be established using Cohen’s conventions, where correlation coefficients of 0.10 are considered small, 0.30 as medium, and 0.50 as large. All correlation coefficients are in the moderate range. As can be seen, a significant positive association was found between emotional labour and emotional exhaustion (*r* = 0.36, *p* < 0.001), whereas there were negative relationships between both emotional support and emotion-focused coping and emotional exhaustion (*r* = −0.34, *p* < 0.001 and *r* = −0.23, *p* < 0.01, respectively). Emotional labour was also inversely associated with emotional support and emotion-focused coping (*r* = −0.22, *p* < 0.01 and *r* = −0.21, *p* < 0.01, respectively).

Hierarchical regression analysis was utilised to examine whether the two potential resources (emotional support and emotion-focused coping) moderated the relationship between emotional labour and emotional exhaustion. Details of both equations are shown in Table 2. Nursing experience (i.e., year of study) was entered in step 1 of each equation to control for its potential effects. At step 2, the standardised scores for emotional labour and each of the potential resources (i.e., emotional support and emotion-focused coping) were added to establish their main effects. In step 3, the moderation terms were entered; i.e., (a) emotional labour × emotional support and (b) emotional labour × emotion-focused coping. Support for a moderation effect is found if step 3 accounts for a significant proportion of the variance in the outcome variable. The first equation explained a total of 29 percent of the variance in emotional exhaustion. Job experience (entered in step 1) accounted for 3 percent of the variance (in a positive direction), while emotional labour and emotion-focused coping together explained 23 percent. The interaction term entered in the third and final step of this equation added a further 3 percent to the incremental variance. The second equation accounted for 59 percent of the variance in emotional exhaustion. Job experience explained three percent of the variance, emotional labour and emotional support accounted for 54 percent, and the interaction term explained a further two percent.

**Figure 1:**
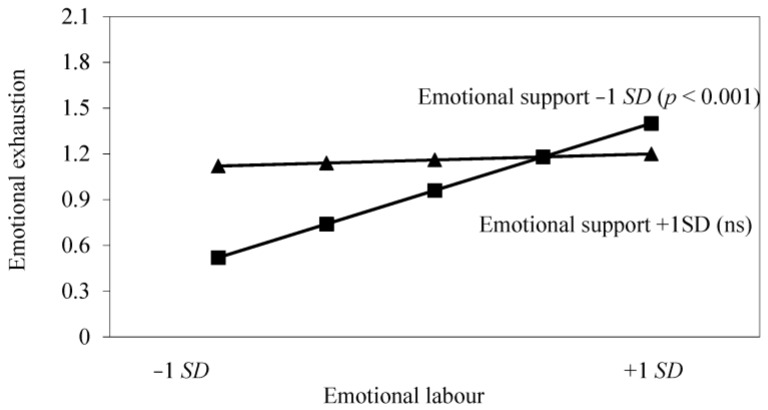
Interaction effect of emotional labour and emotional support on emotional exhaustion.

**Figure 2:**
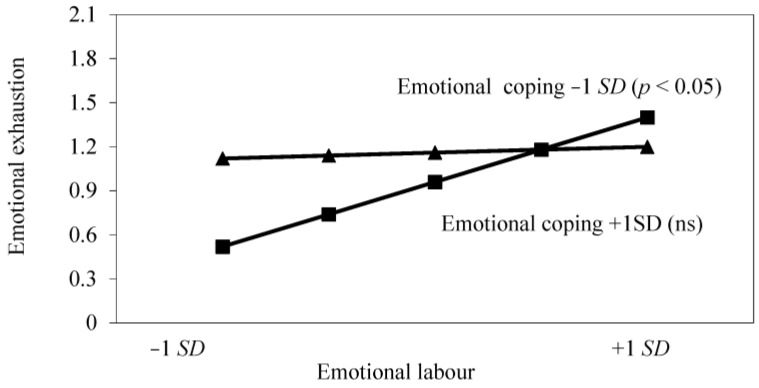
Interaction effect of emotional labour and emotional-focused coping on emotional exhaustion.

Figure 1 and Figure 2 plot the relationships between emotional labour and emotional exhaustion for high and low levels of each of the potential resources; i.e., one standard deviation above and below the standardised mean. As can be seen from Figure 1, evidence was found that emotional support moderated the relationship between emotional demands and emotional exhaustion (*p* < 0.001). Where emotional support was low, an increase in emotional labour was associated with an increase in emotional exhaustion, but these two variables were not significantly related under conditions of high emotional support. Figure 2 also provides evidence that emotion-focused coping moderates the relationship between emotional labour and emotional exhaustion, whereby an increase in emotional labour was associated with more emotional exhaustion where emotion coping was low, but emotional labour was not related to this outcome under conditions of high emotion-focused coping.

## 4. Discussion

This study has provided insight into the emotional labour performed by student nurses and the resources that can protect them from its negative effects. Nurses who performed more emotional labour at work reported a higher level of emotional exhaustion, whereas those who were able to draw upon more emotional resources—in terms of emotional support and opportunities for venting emotion—were less likely to be emotionally exhausted. Some support for the triple-match hypothesis was also found in that both emotional support and emotionally expressive coping moderated the positive association between emotional labour and emotional exhaustion. These findings have the potential to provide an evidence base for interventions to help nurses manage the emotional demands of their work.

It might be assumed that the drive to build a culture of compassionate, patient-centred care in the UK will improve the patient experience [10]. Although this is a worthwhile aim, it is crucial for policy makers to consider the risks faced by staff in delivering high quality compassionate care. In accordance with the findings of previous research [25,32], this study has highlighted a strong link between the emotional demands of the caring role and feelings of emotional depletion. Emotional exhaustion will not only affect the wellbeing of healthcare staff, but more indirectly influence the quality of patient care via outcomes such as compassion fatigue [11]. Research has found strong links between burnout in nurses and a range of negative outcomes such as patient satisfaction, safety climate, and mortality rates, as well as recruitment and retention [62,63,64].

The finding that levels of emotional exhaustion increased over time is a particular cause for concern. Interventions at an early stage in nursing education are vital in order to help early career staff develop strategies to provide compassionate care to patients and their families without sacrificing their own wellbeing. It is recognised that initial nursing education and subsequent professional development opportunities do not place sufficient focus on the requirements of delivering compassionate care [9]. The need for an “emotional curriculum” has been emphasised that not only provides nurses with clinical knowledge and skills, but also has caring and compassionate values at its heart [46,65]. A key aim of this curriculum should be to highlight the risks of emotional labour in the profession and the need to develop emotional resilience, and associated competencies (such as “compassion literacy”) in order to manage the emotional demands of practice in a healthy and sustainable way [66,67].

The findings of this study highlight the importance of emotional support and opportunities for emotional venting in helping student nurses meet the emotional challenges of practice. In terms of the clinical working environment, there is evidence that a strong and supportive team culture can help nurses manage the emotional demands of their role and protect them against burnout [68]. There is also evidence that a climate of authenticity where staff are encouraged to take a “self-regulatory” break from emotionally-charged interactions with patients can protect them against the negative impact of emotional labour [28]. A novel approach to enhancing compassion literacy is offered by Winch and colleagues [69], indicating that a “clinical compassion café” environment can provide informal opportunities for staff to create space for reflection, discuss emotional reactions to practice, and reaffirm core nursing values.

Social support is one of the most effective stress management resources. It can also foster feelings of social connectedness and protect against social alienation and burnout [46,70]. The findings of this study suggest that interventions that aim to enhance emotional support would be particularly successful in helping student nurses manage emotional labour. There is evidence that mentoring programmes can offer social support in healthcare environments and can develop emotional competencies such as empathy in inexperienced practitioners [71,72]. Mutually supportive relationships with peers may be a particularly important resource in helping student nurses meet the emotional challenges of practice [73]. Peer coaching involves the creation of a collaborative and reciprocal relationship with a colleague that is characterised by mutual trust and concern [74]. This technique can facilitate a solution-focused approach to problems and protect personal resources and wellbeing during stressful periods [75]. Peer coaching also has strong potential to help nurses explore different perspectives on emotional interactions with patients and their families and explore strategies for providing compassionate care while protecting their personal wellbeing. The use of role-playing techniques during peer coaching sessions could also enable nurses to practice empathic responses and prepare for emotionally-charged interactions.

The findings of this study suggest that opportunities for venting emotions within trusting and non-judgemental relationships would help student nurses manage emotional labour more effectively. It is acknowledged, however, that such relationships may not be easily developed, especially in a working environment where high demands and long antisocial hours are the norm. There is evidence that emotional writing interventions can help people gain awareness of complex emotional experiences and how best to manage them [76]. Guided reflective writing is a form of coping that has been found to facilitate emotional expression and build emotional literacy, empathy, and compassion in health and social care professionals, protecting their mental and physical health [77,78,79]. Such techniques are straightforward and low-cost, and can be particularly useful when opportunities to share and process complex emotional reactions to practice with others are limited.

More research is required to help nurses more effectively manage the emotional demands of practice. Studies with longitudinal designs should follow nurses through their training and preceptorship period to gain insight into their emotional socialisation into the profession, how compassionate care is shaped over time, and the role played by support and coping styles in predicting outcomes such as compassion fatigue, compassion satisfaction, and burnout. Insight is also needed into how nurses make empathic connections and construct emotional boundaries when interacting with patients and their families and the implications for their wellbeing over time. A diary study conducted by Donoso and colleagues [32] found that the emotional regulation strategies utilised by nurses underpin their reactions to the emotional demands of practice. In order to develop interventions to help nurses manage emotional interactions more effectively and avoid burnout, insight is needed into the type of emotion regulation strategies that can protect or threaten wellbeing and patient satisfaction.

A measure of compassion competence for nurses has been recently developed in order to identify the relevant knowledge, skills, and attitudes required to deliver compassionate care [80]. The scale encompasses the ability to express understanding and compassion towards patients and their families, recognise and react to changes in patients’ emotions, and gain insight into patients’ emotional needs. While these factors are crucial in enhancing the patient experience, the findings of the present study also emphasise the importance of assessing nurses’ capacity for self-care and the effective utilisation of emotional regulation strategies to help them replenish their emotional resources.

This study has extended knowledge of the emotional demands experienced by student nurses and the implications for their wellbeing; insight has also been gained into the individual and contextual factors that might be protective. Nonetheless, its cross-sectional and correlational design means that causality cannot be established. The findings reported in this paper are likely to reflect a dynamic process whereby the emotional demands experienced by student nurses will impact their wellbeing, which in turn will shape their interactions with patients and their families. As discussed above, daily diary research has strong potential to elucidate this unfolding process and identify the type of emotional demands and the individual and contextual factors that can protect and threaten the wellbeing of nurses. A further limitation to this study is that respondents were predominantly white British. There is evidence that students’ experiences of emotional labour and the role played by social support and coping is subject to cultural differences [50,52]; therefore, the extent to which research findings are applicable to a culturally diverse workforce should be further examined.

## 5. Conclusions

This study provides evidence that student nurses experience emotional labour that can affect their wellbeing. It has identified resources that can help them manage the emotional demands of practice more effectively and provide their patients with high quality compassionate care. A range of interventions with the potential to enhance compassion literacy and reduce the negative impact of emotional labour have been identified.

## Figures and Tables

**Table 1:** Correlations between study variables.

Study Variables	*M*	*SD*	1	2	3	4
1. Emotional labour	3.36	0.46	1.0			
2. Emotional exhaustion	4.24	0.96	0.36 ***	1.0		
3. Emotional support	3.93	0.68	−0.22 **	−0.34 ***	1.0	
4. Emotion-focused coping	2.04	0.41	−0.21 **	−0.23 **	0.24 **	1.0

Note: ** *p* < 0.01, *** *p* < 0.001.

**Table 2:** Moderating effects of emotional support and emotion-focused coping.

**Emotional Support as Moderator between Emotional Labour and Emotional Exhaustion**			
**Predictor**	**Step 1**	**Step 2**	**Step 3**
Job experience	0.02 **	0.01	0.01
Emotional labour		0.20 **	0.32 **
Emotion-focused coping		−0.23 ***	−0.21 **
Interaction (a × b)			0.22 **
Total *R*^2^	0.03 **	23 ***	0.03 **
**Emotion-Focused Coping as Moderator between Emotional Labour and Emotional Exhaustion**			
**Predictor**	**Step 1**	**Step 2**	**Step 3**
Job experience	0.02 **	0.01 *	0.01 *
Emotional labour		0.43 ***	0.16 **
Emotional support		−0.35 ***	−0.63 ***
Interaction (a × b)			0.18 **
Total *R*^2^	0.03 ***	0.54 ***	0.02 **

Note: * *p* < 0.05; ** *p* < 0.01; *** *p* < 0.001; Standardised coefficients are reported.
